# Trends in cardiovascular mortality among lung cancer patients in the United States: a retrospective study from 1999 to 2023

**DOI:** 10.1186/s40959-026-00512-z

**Published:** 2026-05-22

**Authors:** Yong-li Chen, Jian-quan Chen, Lin-wen Zeng, Jin-yan Wu, Xiao‐xia Qiu, Zhen-nan Lin, Jie Xiao, Xing Miao, Re‐hua Wang, Jian‐cheng Zhang

**Affiliations:** 1 Department of Cardiology, Fuzhou University Affiliated Provincial Hospital, Shengli Clinical Medical College of Fujian Medical University, Fujian Provincial Hospital, Fuzhou, Fujian 350000 PR China; 2 Department of Cardiology, Fujian Medical University Union Hospital, Fuzhou, Fujian 350000 PR China; 3 Fujian Provincial Key Laboratory of Cardiovascular Disease, Fujian Cardiovascular Institute, Fujian Provincial Center for Geriatrics, Fujian Clinical Medical Research Center for Cardiovascular Diseases, Fuzhou, Fujian 350000 PR China; 4 Department of Ultrasound, the First Affiliated Hospital, Fujian Medical University, Fuzhou, Fujian 350005 PR China; 5 Department of Ultrasound, Binhai Campus of the First Affiliated Hospital, National Regional Medical Center, Fujian Medical University, Fuzhou, Fujian 350212 PR China

**Keywords:** Lung cancer, Cardiovascular disease, Health disparities, Mortality trend reversal, Population-based study, Joinpoint regression

## Abstract

**Background:**

Although cardiovascular disease (CVD) frequently co-occurs with lung cancer and adversely affects prognosis, the temporal patterns of CVD mortality in this patient group remain inadequately explored. We aimed to clarify trends and disparities in CVD mortality among United States (US) adults with lung cancer.

**Methods:**

Utilizing the Centers for Disease Control and Prevention’s Wide-Ranging Online Data for Epidemiologic Research (CDC WONDER) spanning from 1999 to 2023 for adults aged 35 years and older, we calculated age-adjusted mortality rates (AAMRs) and crude mortality rates (CMRs) per 100,000 individuals, with the AAMRs standardized to the 2000 US population. The annual percent change (APC) and average annual percent change (AAPC) were estimated using joinpoint regression analysis. In CDC WONDER, the National Center for Health Statistics assigns the underlying cause of death using standardized World Health Organization rules. Thus, we defined CVD as the underlying cause of death and lung cancer as a contributing cause based on the database’s coding framework rather than investigator judgment.

**Results:**

Among 93,859 CVD deaths in lung cancer patients, mortality declined significantly from 1999 to 2015 (APC: − 4.70), but increased in females from 2015 to 2023 (APC: 1.73), while remaining stable in males (APC: 0.44). Males experienced a more pronounced reduction in AAMRs, decreasing from 5.39 in 1999 to 2.24 in 2023, but consistently had higher CVD mortality rates than females. During most of the study period, Non-Hispanic African American or Black (NH Black) individuals experienced the highest AAMRs, followed by NH Whites. Long-term decline in CVD mortality reversed significantly among individuals aged ≥ 85 years since 2017. Significant differences in AAPC relative to the general population were identified among the Northeast, NH Black, NH White, males, and across all age groups except for the 75–84 cohort.

**Conclusions:**

Although CVD mortality in the US declined from 1999 to 2023, this trend has stalled or reversed among adults with lung cancer since 2015. Ongoing disparities by sex, race, age, region, and state emphasize the urgent need for targeted interventions for these vulnerable groups.

**Supplementary Information:**

The online version contains supplementary material available at 10.1186/s40959-026-00512-z.

## Highlights

Long-term decline in cardiovascular disease (CVD) mortality reversed among females and Non-Hispanic White individuals with lung cancer since 2015.

Males had consistently higher CVD mortality than females among those with lung cancer.

Non-Hispanic Black individuals had the highest mortality across racial groups for most of the study period.

Long-term decline in CVD mortality reversed significantly among adults aged 85 and older since 2017.

Recent increases in CVD mortality among US adults with lung cancer were observed in the South and Midwest census regions.

**Supplementary Information:**

The online version contains supplementary material available at 10.1186/s40959-026-00512-z.

## Introduction

Cardiovascular disease (CVD), which includes heart disease and stroke, and lung cancer are leading causes of mortality in the United States (US) [1, 2], and they frequently co-occur sharing common risk factors such as smoking, hypertension, diabetes, advanced age, and obesity [3]. Among cancer patients, individuals with lung cancer exhibit the highest prevalence of pre-existing CVD and face the greatest risk of developing cardiovascular events following their cancer diagnosis [4], while CVD may contribute to both the incidence and the increased mortality associated with lung cancer [5].

With ongoing advancements in cancer therapies, the survival rates of lung cancer patients have markedly improved. Since 2015, the therapeutic landscape for non-small cell lung cancer (NSCLC) has undergone substantial transformation due to the extensive adoption of targeted therapies and immune checkpoint inhibitors (ICIs). In particular, targeted treatments for ALK-positive NSCLC have emerged as the new standard of care [6]. Meanwhile, programmed cell death protein 1 (PD-1) inhibitors like nivolumab and pembrolizumab were quickly approved for treating both squamous and nonsquamous lung cancer in previously treated patients. These ICIs significantly enhance survival rates and have become the leading immunotherapy, transforming the treatment approach for advanced lung cancer [7, 8], while ICIs are well-documented to cause adverse cardiovascular events, including myocarditis, arrhythmias, and accelerated atherosclerosis [9, 10].

A thorough examination of long-term trends in CVD mortality among adults diagnosed with lung cancer is relatively limited in the existing literature. Previous research has frequently been constrained by limited sample sizes and a tendency to treat lung cancer and CVD as distinct entities [11–13] rather than interconnected comorbidities, or have focused on narrow subsets of cardiac disease [14]. No previous study has compared the average annual percent change (AAPC) in CVD mortality between lung cancer patients and the general US population.

The existing body of literature reveals that between 1999 and 2019, Black women and men in the US persistently exhibited higher cardiovascular mortality rates compared to their White counterparts [15]. Furthermore, racial and geographic disparities significantly impact lung cancer-related mortality. Research indicates that, despite an overall decline in lung cancer mortality rates, African Americans and individuals residing in non-metropolitan areas continue to experience elevated mortality rates [16, 17]. These disparities underscore the necessity of incorporating a range of socioeconomic and geographic factors in mortality trend analyses. Nevertheless, the current literature generally lacks stratified analyses of long-term trends in cardiovascular mortality among lung cancer populations, particularly with respect to key demographic variables such as age, sex, race, and geographic region.

To address this gap, we conducted a retrospective analysis using the CDC WONDER Multiple Cause of Death database spanning 1999 to 2023. We systematically examined temporal trends in CVD mortality among US adults with lung cancer, with stratification by sex, race/ethnicity, age group, census region, and state, to identify subpopulations with disproportionately high or shifting mortality burdens and to inform targeted public health and clinical interventions in cardio-oncology.

## Methods

### Study design

The Multiple Cause of Death database from CDC WONDER was utilized to acquire death certificate data spanning from 1999 to 2023 for individuals aged 35 years and older in the US. CVD mortality was identified using International Classification of Diseases (ICD)–10 codes I00–I99 as the underlying cause of death, while lung cancer was recorded as a contributing cause using ICD–10 codes C34.0–C34.3 and C34.8–C34.9 [18]. As the dataset comprised de-identified public use data, obtaining approval from an institutional review board was deemed unnecessary. The study adhered to the STROBE (Strengthening the Reporting of Observational Studies in Epidemiology) guidelines [19].

### Data abstraction

The extracted variables encompassed population size, sex, race/ethnicity, year, region, and state. Race/ethnicity was categorized as non-Hispanic (NH) White, NH Black or African American, Hispanic or Latino, and NH Other (from 1999 to 2020, this included “American Indian or Alaska Native” and “Asian or Pacific Islander”; from 2021 to 2023, the categories expanded to include “American Indian or Alaska Native,” “Asian,” “Native Hawaiian or Other Pacific Islander,” and “More than one race.“) Census regions were delineated as Midwest, Northeast, South, and West, following the standardized definitions provided by the US Census Bureau [20]. Joinpoint regression analysis was conducted using AAMRs (per 100,000 population, standardized to the 2000 US population) for all analyses, including subgroup analyses by sex, race/ethnicity, age group, census region, and state.

### Statistical analysis

Trends in CVD mortality with lung cancer were assessed using annual percentage change (APC) and 95% confidence interval (CI). Data from the District of Columbia, Wyoming, Alaska, Utah, and individuals aged 35–44 years were excluded due to unreliable data, defined as instances in which the death count was less than 20 in a given year [21]. APCs, along with average annual percent change (AAPC) and their corresponding CIs, were calculated using the Joinpoint Regression Program (version 5.1.0.0) [22]. This analysis identified significant variations in AAMRs over time by employing a log-linear regression model [23]. A two-tailed t-test was utilized to determine the statistical significance of mortality variation from baseline, with a p-value of less than 0.05 considered significant. For trend analysis, the statistical significance of joinpoints and APCs was evaluated using Monte Carlo permutation tests, adhering to an overall significance level of 0.05, as per the default settings of the Joinpoint Regression Program (National Cancer Institute). Furthermore, the statistical significance of the disparity in the AAPC in CVD mortality rates between lung cancer patients and the general population was assessed using z-tests.

## Results

**Fig. 1:**
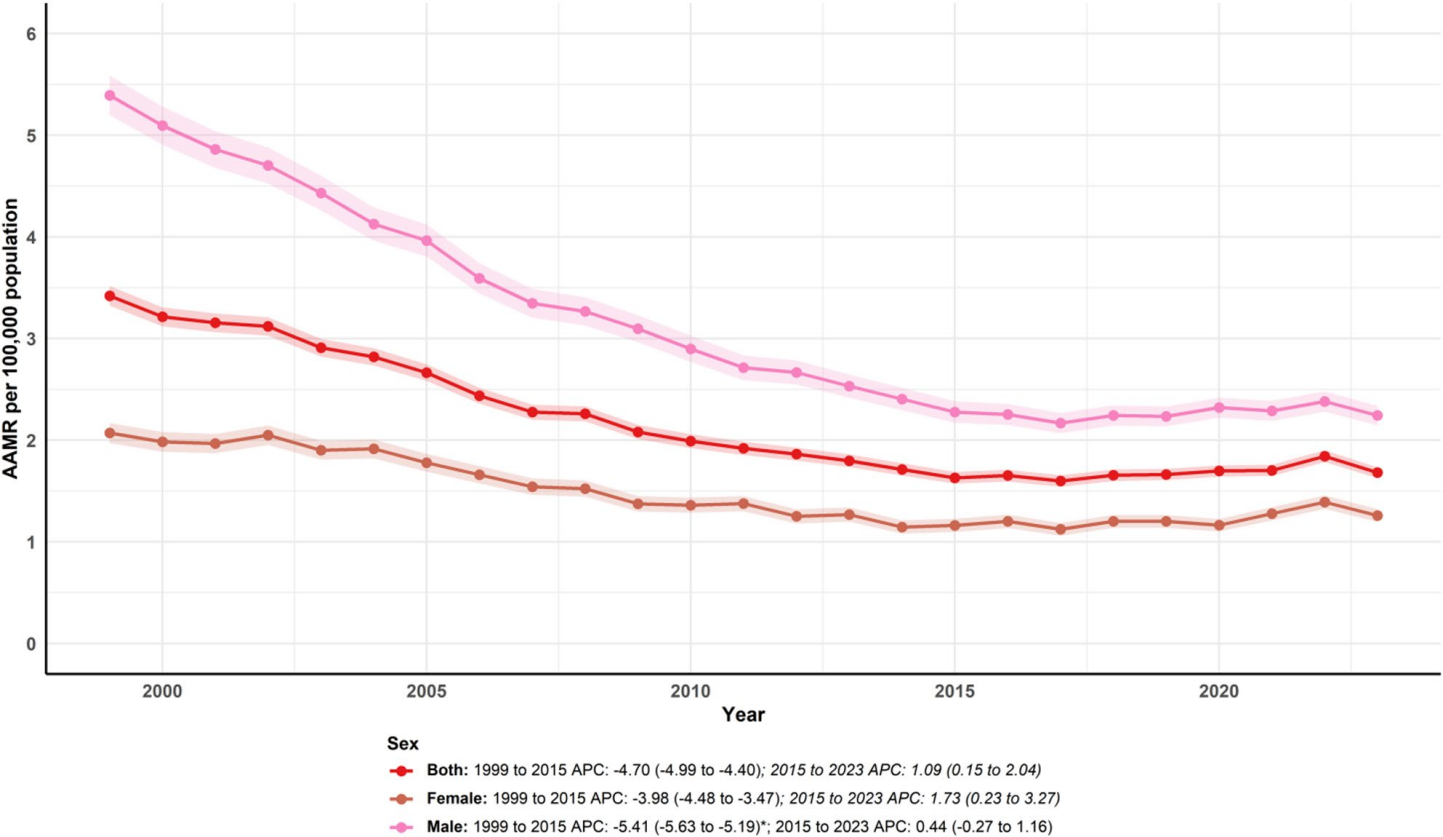
Age-adjusted mortality rate (AAMR) categorized by sex in cardiovascular disease mortality among adults with lung cancer in the US, 1999–2023

Between 1999 and 2023, there were 93,859 recorded cases of CVD as the underlying cause of death among adults with lung cancer in the US (Supplementary Table S4). Throughout this period, the number of deaths decreased by 20.59%, alongside a notable decline in the AAMRs from 3.42 to 1.68 per 100,000 population (Table 1). The mortality trend of CVD among adults with lung cancer showed a significant decline from 1999 to 2023, with an AAPC of − 2.11* (95% CI: − 2.67 to − 1.55) for females, compared to − 3.50* (95% CI: − 3.76 to − 3.24) for males (Fig. 1; Table 2).

**Table 1:** Demographic characteristics of deaths and age-adjusted mortality rates for cardiovascular disease with lung cancer in 1999 and 2023

Characteristic	Deaths			AAMR		
	1999	2023	Percent Change (%)	1999(95% CI)	2023(95% CI)	Decreased values
Sex						
Both	4,817	3,825	–20.59	3.42 (3.32 to 3.52)	1.68 (1.63 to 1.73)	–1.74
Female	1,736	1,584	–8.76	2.07 (1.97 to 2.17)	1.26 (1.20 to 1.32)	–0.81
Male	3,081	2,241	–27.26	5.39 (5.20 to 5.59)	2.24 (2.15 to 2.34)	–3.15#
Census Region						
Midwest	1,188	952	–19.87	3.61 (3.40 to 3.81)	1.99 (1.86 to 2.12)	–1.62
Northeast	1,108	721	–34.93	3.74 (3.52 to 3.96)	1.71 (1.59 to 1.84)	–2.03#
South	1,654	1,465	–11.43	3.33 (3.17 to 3.49)	1.69 (1.60 to 1.78)	–1.64
West	867	687	–20.76	3.10 (2.89 to 3.31)	1.36 (1.25 to 1.46)	–1.74
Race						
Hispanic	81	147	81.48	1.28 (1.01 to 1.60)	0.70 (0.59 to 0.82)	–0.58
NH Black^a^	448	434	–3.12	3.79 (3.44 to 4.15)	1.95 (1.76 to 2.14)	–1.84#
NH Other^b^	71	139	95.77	1.93 (1.49 to 2.46)	0.92 (0.77 to 1.08)	–1.01
NH White	4,207	3,092	–26.50	3.57 (3.46 to 3.68)	1.85 (1.79 to 1.92)	–1.72
Age Groups^c^						
45 to 54 years	140	51	–63.57	0.38 (0.32 to 0.45)	0.13 (0.09 to 0.17)	–0.25
55 to 64 years	582	448	–23.02	2.45 (2.25 to 2.65)	1.07 (0.97 to 1.17)	–1.38
65 to 74 years	1,567	1,188	–24.19	8.51 (8.09 to 8.93)	3.43 (3.23 to 3.62)	–5.08
75 to 84 years	1,859	1,428	–23.18	15.21 (14.52 to 15.90)	7.77 (7.37 to 8.18)	–7.44#
85 + years	649	703	8.32	15.62 (14.42 to 16.83)	11.35 (10.51 to 12.19)	–4.27

*Abbreviations*: *AAMR* Age-adjusted mortality rate, *CI* Confidence interval, *NH* Not Hispanic or Latino

^a^NH Black, African American or Black

^b^NH Other: from 1999 to 2020, includes “American Indian or Alaska Native” and “Asian or Pacific Islander.” From 2021 to 2023, it includes four categories: “American Indian or Alaska Native,” “Asian,” “Native Hawaiian or Other Pacific Islander,” and “More than one race”

^c^Crude Mortality Rate is used for analysis instead of age-adjusted mortality rates for Age groups

#The most pronounced AAMR reduction within their respective groups

**Table 2:** Annual percentage changes and average annual percentage changes in cardiovascular disease mortality with lung cancer, 1999 to 2023

Variable	Trend Segment	Start	End	APC (95% CI)	*P*-value	AAPC (95% CI)	*P*-value
Overall	1	1999	2015	–4.70*(–4.99to–4.40)	<0.000001	–2.81*(–3.15to–2.46)	< 0.000001
	2	2015	2023	1.09*(0.15to2.04)	0.024803		
Census Region							
Midwest	1	1999	2005	–3.59*(–4.66to–2.52)	0.000006	–2.26*(–2.94to–1.56)	< 0.000001
	2	2005	2010	–6.23*(–8.42to–4.00)	0.000042		
	3	2010	2016	–2.05*(–3.76to–0.31)	0.024384		
	4	2016	2023	1.70*(0.70to2.71)	0.002600		
Northeast	1	1999	2017	–4.14*(–4.46to–3.83)	<0.000001	–3.10*(–3.59to–2.61)	< 0.000001
	2	2017	2023	0.10(–1.80to2.04)	0.911435		
South	1	1999	2014	–5.06*(–5.56to–4.56)	<0.000001	–2.64*(–3.14to–2.14)	< 0.000001
	2	2014	2023	1.53*(0.36to2.71)	0.012686		
West	1	1999	2015	–5.26*(–5.71to–4.81)	<0.000001	–3.41*(–3.93to–2.89)	< 0.000001
	2	2015	2023	0.40(–1.03to1.85)	0.569182		
Race							
Hispanic	1	1999	2023	–2.06*(–3.18to–0.93)	0.001035	–2.06*(–3.18to–0.93)	0.001035
NH Black	1	1999	2015	–4.80*(–5.34to–4.25)	<0.000001	–2.81*(–3.42to–2.20)	< 0.000001
	2	2015	2023	1.28(–0.37to2.96)	0.122838		
NH Other	1	1999	2016	–5.17*(–6.63to–3.68)	0.000001	–3.13*(–4.74to–1.49)	0.000207
	2	2016	2023	2.02(–2.77to7.05)	0.396219		
NH White	1	1999	2015	–4.51*(–4.79to–4.23)	<0.000001	–2.59*(–2.91to–2.26)	< 0.000001
	2	2015	2023	1.38*(0.48to2.29)	0.004527		
Sex							
Female	1	1999	2015	–3.98*(–4.48to–3.47)	<0.000001	–2.11*(–2.67to–1.55)	< 0.000001
	2	2015	2023	1.73*(0.23to3.27)	0.026192		
Male	1	1999	2015	–5.41*(–5.63to–5.19)	<0.000001	–3.50*(–3.76to–3.24)	< 0.000001
	2	2015	2023	0.44(–0.27to1.16)	0.212637		
Age Groups							
45 to 54 years	1	1999	2023	–3.74*(–4.41to–3.06)	<0.000001	–3.74*(–4.41to–3.06)	< 0.000001
55 to 64 years	1	1999	2011	–7.06*(–7.90to–6.22)	<0.000001	–3.43*(–4.02to–2.83)	< 0.000001
	2	2011	2023	0.35(–0.61to1.31)	0.457650		
65 to 74 years	1	1999	2015	–5.99*(–6.31to–5.67)	<0.000001	–3.80*(–4.16to–3.43)	< 0.000001
	2	2015	2023	0.75(–0.26to1.77)	0.137676		
75 to 84 years	1	1999	2015	–4.23*(–4.56to–3.90)	<0.000001	–2.69*(–3.07to–2.31)	< 0.000001
	2	2015	2023	0.47(–0.57to1.52)	0.359433		
85 + years	1	1999	2017	–2.82*(–3.37to–2.28)	<0.000001	–1.03*(–1.78to–0.27)	0.007758
	2	2017	2023	4.56*(1.70to7.49)	0.003123		

*Abbreviations*: *APC* Annual percent change, *CI* Confidence interval, *AAPC* Average annual percent change

*Shows that the APC or AAPC significantly deviates from zero at the alpha = 0.05 level

### Trends by sex

Males diagnosed with lung cancer had higher CVD mortality rates than females from 1999 to 2023, and they experienced the most significant reduction in AAMR, declining from 5.39 (95% CI: 5.20 to 5.59) in 1999 to 2.24 (95% CI: 2.15 to 2.34) in 2023. The overall AAPC was − 3.50* (95% CI: − 3.76 to − 3.24), as illustrated in Fig. 1 and detailed in Tables 1 and 2. From 1999 to 2015, mortality trends significantly decreased for both males (APC: − 5.41*; 95% CI: − 5.63 to − 5.19) and females (APC: − 3.98*; 95% CI: − 4.48 to − 3.47). However, during the subsequent period (2015 to 2023), female mortality exhibited a significant increase (APC: 1.73*; 95% CI: 0.23 to 3.27), while the trend for males showed a non-significant increase (APC: 0.44; 95% CI: − 0.27 to 1.16), as depicted in Fig. 1; Table 2. The decline in CVD mortality was significantly more pronounced in adults with lung cancer compared to those with CVD alone among males from 1999 to 2023 (*p* < 0.000001; AAPC: − 3.50*; 95% CI: − 3.76 to − 3.24 for CVD with lung cancer versus AAPC: − 2.03*; 95% CI: − 2.46 to − 1.59 for CVD alone) (Table 3).

**Table 3:** Evaluating the differences in cardiovascular disease mortality between adults with lung cancer and the general US population from 1999 to 2023

Measure	Cardiovascular Disease in Patients with Lung cancer		Overall Cardiovascular Disease-Related Mortality Rates		*P*-value for AAPC Comparison
	AAPC (95% CI)	*P*-Value	AAPC (95% CI)	*P*-value	
Census Region					
Midwest	–2.26*(–2.94to–1.56)	< 0.000001	–1.85*(–2.34to–1.37)	< 0.000001	0.340687
Northeast#	–3.10*(–3.59to–2.61)	< 0.000001	–2.36*(–2.53to–2.19)	< 0.000001	0.005166
South	–2.64*(–3.14to–2.14)	< 0.000001	–1.98*(–2.61to–1.35)	< 0.000001	0.107758
West#	–3.41*(–3.93to–2.89)	< 0.000001	–2.06*(–2.63to–1.48)	< 0.000001	0.000642
Race					
Hispanic	–2.06*(–3.18to–0.93)	0.001035	–2.39*(–3.04to–1.73)	< 0.000001	0.619291
NH Black#	–2.81*(–3.42to–2.20)	< 0.000001	–1.95*(–2.24to–1.67)	< 0.000001	0.012297
NH Other	–3.13*(–4.74to–1.49)	0.000207	–2.59*(–3.08to–2.11)	< 0.000001	0.532550
NH White#	–2.59*(–2.91to–2.26)	< 0.000001	–1.88*(–2.09to–1.66)	< 0.000001	0.000355
Sex					
Female	–2.11*(–2.67to–1.55)	< 0.000001	–2.12*(–2.60to–1.64)	< 0.000001	0.978800
Male#	–3.50*(–3.76to–3.24)	< 0.000001	–2.03*(–2.46to–1.59)	< 0.000001	<0.000001
Age Groups					
45 to 54 years#	–3.74*(–4.41to–3.06)	< 0.000001	–0.85*(–1.41to–0.29)	0.003219	<0.000001
55 to 64 years#	–3.43*(–4.02to–2.83)	< 0.000001	–1.24*(–1.57to–0.91)	< 0.000001	<0.000001
65 to 74 years#	–3.80*(–4.16to–3.43)	< 0.000001	–2.29*(–2.78to–1.81)	< 0.000001	0.000001
75 to 84 years	–2.69*(–3.07to–2.31)	< 0.000001	–2.59*(–3.00to–2.17)	< 0.000001	0.727597
85 + years#	–1.03*(–1.78to–0.27)	0.007758	–1.92*(–2.17to–1.66)	< 0.000001	0.028598

*Abbreviations*: *AAPC *Average annual percent change, *CI* Confidence interval

*Indicates that the AAPC is significantly different from zero at the alpha = 0.05 level

#Indicates that the CVD mortality downward trend in adults with lung cancer differed significantly from that of CVD alone at the alpha = 0.05 level

### Trends by race

**Fig. 2:**
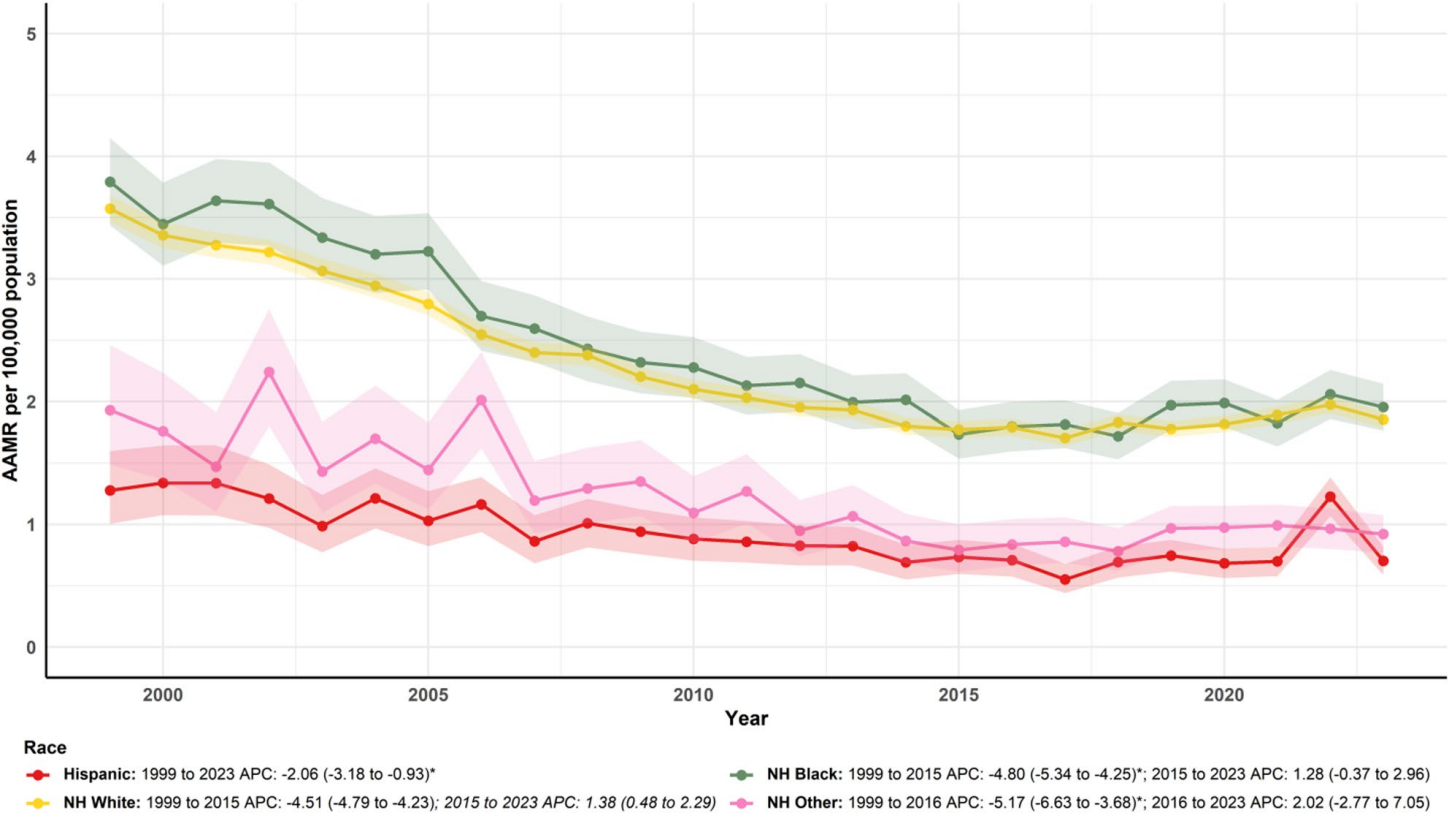
Age-adjusted mortality rate (AAMR) categorized by race in cardiovascular disease mortality among adults with lung cancer in the US, 1999–2023

Changes in the number of deaths show pronounced racial disparities from 1999 to 2023. NH White individuals experienced a 26.50% decrease, while NH Black individuals experienced a 3.12% decrease. In contrast, Hispanic or Latino and NH Other groups exhibited substantial increases of 81.48% and 95.77%, respectively, but their AAMRs remained lower than other racial groups studied. This absolute increase is driven by population growth or demographic shifts rather than increased individual risk (Table 1; Fig. 2). From 1999 to 2023, there were significant reductions in CVD mortality among adults with lung cancer across all racial groups in the US (Table 2). The Non-Hispanic Black population experienced the most pronounced decline in AAMR, decreasing from 3.79 (95% CI: 3.44 to 4.15) in 1999 to 1.95 (95% CI: 1.76 to 2.14) in 2023. The overall average annual percent change (AAPC) was − 2.81* (95% CI: − 3.42 to − 2.20) (Fig. 2; Tables 1 and 2).

Hispanic or Latino individuals experienced a gradual yet consistent decline in mortality rates, characterized by a single-phase decrease (AAPC: − 2.06*; 95% CI: − 3.18 to − 0.93). Between 1999 and 2015, NH White individuals exhibited a significant reduction in mortality (APC: − 4.51*; 95% CI: − 4.79 to − 4.23). However, from 2015 to 2023, there was a notable increase in mortality rates within this group (APC: 1.38*; 95% CI: 0.48 to 2.29). NH Black and NH Other individuals also experienced significant declines in mortality from 1999 to 2015 (APC: − 4.80*; 95% CI: − 5.34 to − 4.25) and from 1999 to 2016 (APC: − 5.17*; 95% CI: − 6.63 to − 3.68), respectively. Nevertheless, these declines plateaued in subsequent years, with non-significant trends observed for NH Black individuals (from 2015 to 2023, APC = 1.28, *p* = 0.12) and NH Other individuals (from 2016 to 2023, APC = 2.02, *p* = 0.40) (Table 2; Fig. 2). The decline in CVD mortality was significantly more pronounced among adults with concurrent lung cancer compared to those with CVD alone, both in NH White (AAPC: − 2.59* vs. − 1.88*; *p* = 0.0004) and NH Black individuals (AAPC: − 2.81* vs. − 1.95*; *p* = 0.01) (Table 3).

### Trends by age groups

**Fig. 3:**
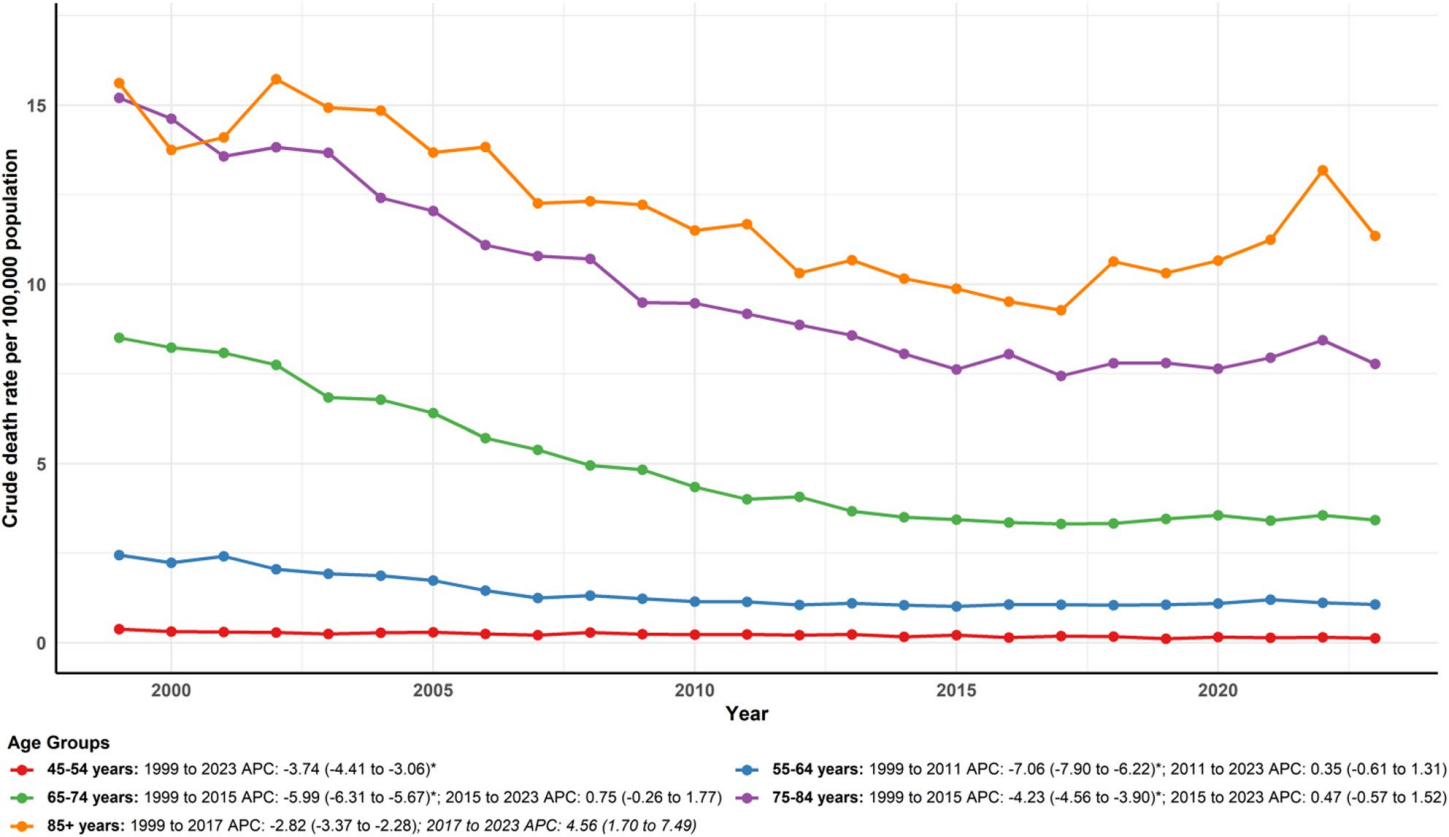
Crude mortality rates (CMRs) categorized by age in cardiovascular disease mortality among adults with lung cancer in the US, 1999–2023

The age groups were categorized as 45–54, 55–64, 65–74, 75–84, and 85 years and older. Notably, the oldest cohort (85 years and older) was the only group to exhibit an increase in the number of deaths from 1999 to 2023, with a rise of 8.32% (Table 1). Figure 3 demonstrates a positive correlation between crude mortality rates (CMRs) and age. The highest CMR was recorded among individuals aged 85 years and older in both 1999 (CMR: 15.62, 95% CI: 14.42 to 16.83) and 2023 (CMR: 11.35, 95% CI: 10.51 to 12.19). This was followed by the 75–84 age group (CMR in 2023: 7.77, 95% CI: 7.37 to 8.18) and the 65–74 age group (CMR in 2023: 3.43, 95% CI: 3.23 to 3.62). Lower mortality rates were observed in the younger age groups, with the 55–64 age group exhibiting a CMR of 1.07 (95% CI: 0.97 to 1.17) in 2023, and the 45–54 age group demonstrating the lowest rate in 2023 (CMR: 0.13, 95% CI: 0.09 to 0.17). A similar declining trend was observed in 2019 (Table 1).

The age group 45–54 exhibited a consistent and significant decline throughout the entire period, with an AAPC of − 3.74* (95% CI: − 4.41 to − 3.06). The age group 55–64 experienced a notably rapid initial decrease in CMR from 1999 to 2011 (APC: − 7.06*; 95% CI: − 7.90 to − 6.22), after which no significant downward trend was observed, culminating in a significant long-term AAPC of − 3.43* (95% CI: − 4.02 to − 2.83). Similarly, individuals aged 65–74 underwent a period of rapid decline in CMR from 1999 to 2015 (APC: − 5.99*; 95% CI: − 6.31 to − 5.67), followed by a stagnation, resulting in an overall AAPC of − 3.80* (95% CI: − 4.16 to − 3.43). For the age group 75–84, a significant decline was observed until 2015 (APC: − 4.23*; 95% CI: − 4.56 to − 3.90), after which no significant downward trend was observed, leading to a long-term AAPC of − 2.69* (95% CI: − 3.07 to − 2.31).

The age group of individuals aged 85 years and older emerges as a critical area of concern. Between 1999 and 2017, this demographic experienced a modest yet statistically significant decline in mortality, with an APC of − 2.82* (95% CI: − 3.37 to − 2.28). However, this trend has markedly reversed in subsequent years. From 2017 to 2023, there has been a significant increase in mortality, with an APC of 4.56* (95% CI: 1.70 to 7.49). The overall long-term AAPC for this age group was − 1.03* (95% CI: − 1.78 to − 0.27). (Fig. 3; Table 2) The decline in CVD mortality rates among adults diagnosed with lung cancer exhibited significant differences compared to those with CVD alone across the 45–54 (*p* < 0.000001), 55–64 (*p* < 0.000001), 65–74 (*p* = 0.000001), 85 years and older (*p* = 0.03) age groups, as illustrated in Table 3. Notably, individuals aged 75–84 experienced the most pronounced reduction in CMR, decreasing from 15.21 (95% CI: 14.52 to 15.90) in 1999 to 7.77 (95% CI: 7.37 to 8.18) in 2023. The overall AAPC was calculated to be − 2.69* (95% CI: − 3.07 to − 2.31). Nevertheless, within this age cohort, the rate of CVD mortality decline was not significantly correlated with the presence of lung cancer (*p* = 0.73) (Fig. 3; Tables 1, 2, and 3).

### Trends by census region

**Fig. 4:**
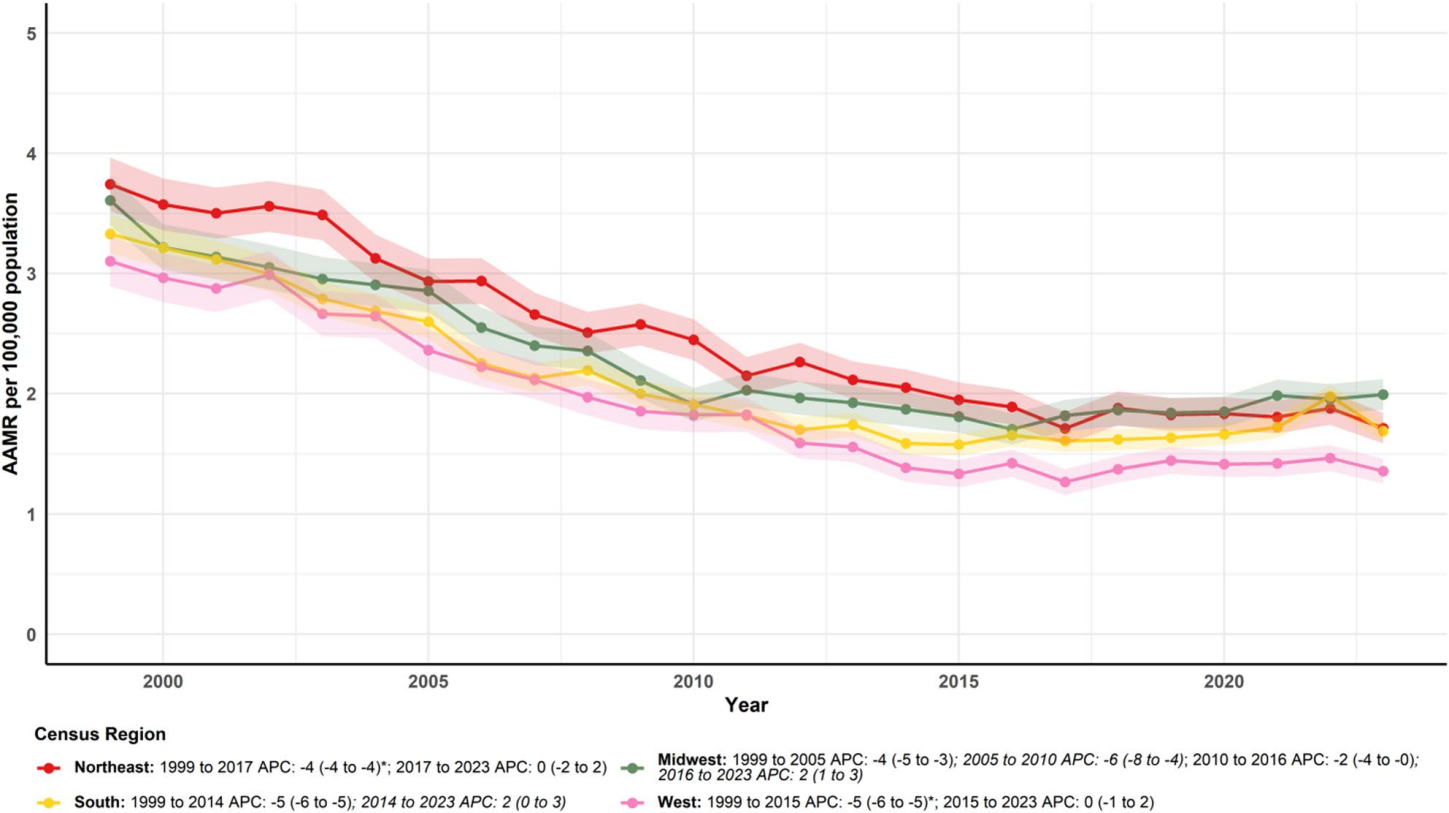
Age-adjusted mortality rate (AAMR) categorized by region in cardiovascular disease mortality among adults with lung cancer in the US, 1999–2023

All regions exhibited a decline in AAMRs. The Northeast experienced the most pronounced percentage reduction in death counts at 34.93%, whereas the South saw an 11.43% decrease (Table 2). The rate of CVD mortality among adults with lung cancer showed a consistent decline across all four regions from 1999 to 2023. Individuals in the Northeast saw the most pronounced reduction in AAMR, decreasing from 3.74 (95% CI: 3.52 to 3.96) in 1999 to 1.71 (95% CI: 1.59 to 1.84) in 2023. The AAPC was − 3.10* (95% CI: − 3.59 to − 2.61) (Fig. 4; Tables 1 and 2).

Mortality trends in the Midwest and South showed similar patterns. The Midwest experienced three phases of significant decline from 1999 to 2016, with APCs ranging from − 2.05* to − 6.23*, followed by a significant increase through 2023 (APC: 1.70*; 95% CI: 0.70 to 2.71). The South saw a significant decline from 1999 to 2014 (APC: − 5.06*; 95% CI: − 5.56 to − 4.56), followed by a significant upward trend thereafter (APC: 1.53*; 95% CI: 0.36 to 2.71). The Northeast experienced a significant decline from 1999 to 2017 (APC: − 4.14*; 95% CI: − 4.46 to − 3.83), with rates stabilizing until 2023 (APC: 0.10; 95% CI: − 1.80 to 2.04). In a similar trend, mortality rates in the Western region exhibited a marked decline from 1999 to 2015 (APC: − 5.26*; 95% CI: − 5.71 to − 4.81), followed by a period of stabilization extending through 2023 (APC: 0.40; 95% CI: − 1.03 to 1.85) (Table 2). The reduction in CVD mortality was notably more pronounced in adults diagnosed with lung cancer compared to those with CVD alone, across both the Northeast (AAPC: − 3.10* versus − 2.36*; *p* = 0.005) and Western populations (AAPC: − 3.41* versus − 2.06*; *p* = 0.0006) (Table 3).

### Trends by states

**Fig. 5:**
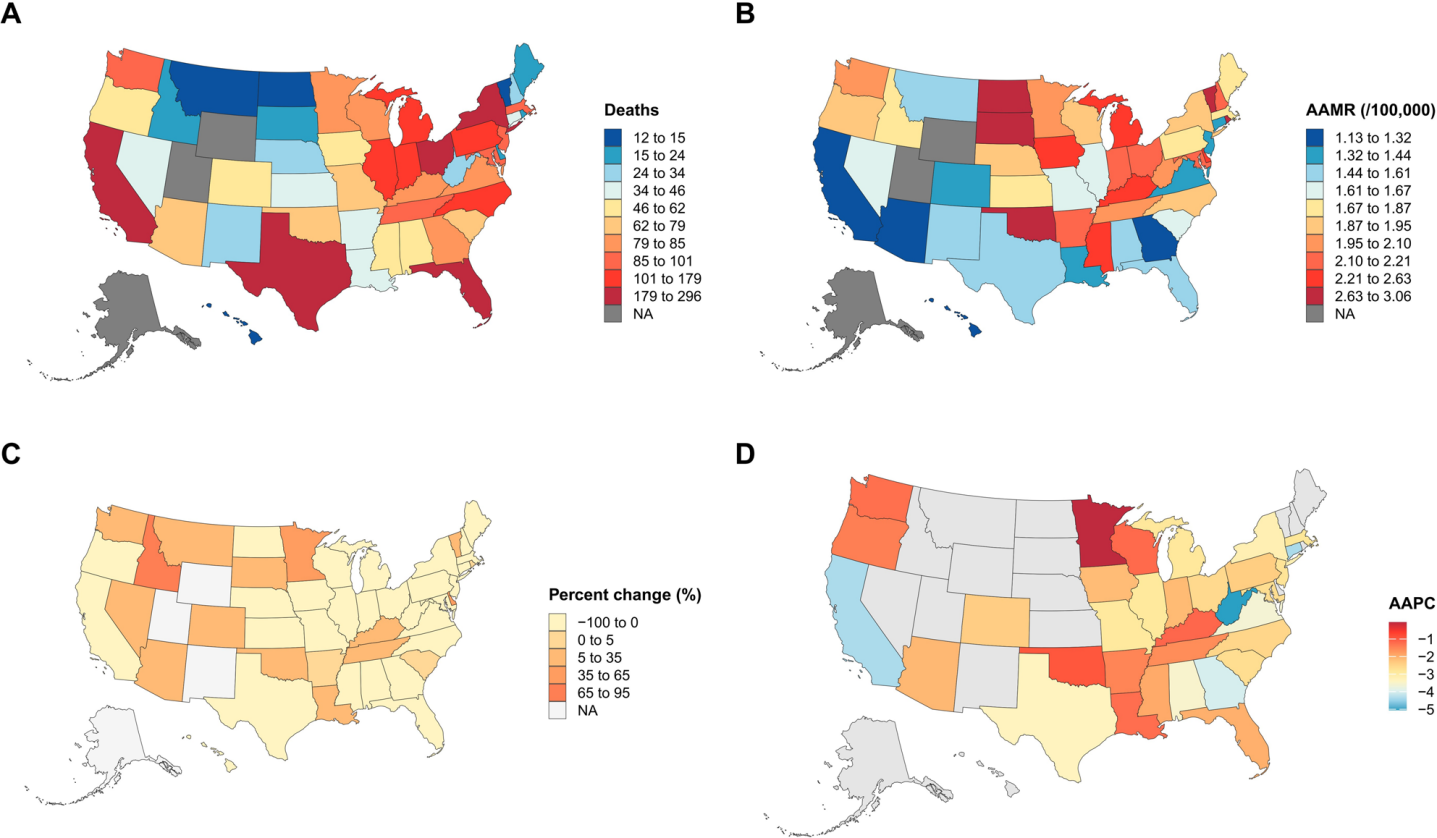
**A** The number of cardiovascular disease deaths among lung cancer patients by state in the US, 2023. **B** State-specific age-adjusted mortality rates (AAMRs) for cardiovascular disease among lung cancer patients, 1999–2023. **C** State-wise percentage change in the number of cardiovascular disease deaths among lung cancer patients, 1999–2023. **D** State-specific average annual percent changes (AAPCs) in mortality from cardiovascular disease among lung cancer patients, 1999 − 2023

Figure 5 illustrates the state-level burden and temporal trends of cardiovascular disease mortality among lung cancer patients in the US. In 2023, the absolute number of cardiovascular deaths varied substantially across states (Fig. 5A). State-specific AAMRs from 1999 to 2023 also demonstrated marked geographic variation (Fig. 5B). The percentage change in the number of cardiovascular deaths from 1999 to 2023 (Fig. 5C) and the AAPC in mortality over the same period (Fig. 5D) revealed heterogeneous temporal trends across states (Fig. 5). From 1999 to 2023, states such as Connecticut and West Virginia experienced pronounced percentage reductions in death counts, at 59.78% and 61.73%, respectively. In contrast, Minnesota reported a 64% increase in the number of deaths during the same period (Supplementary Table S1). West Virginia, which recorded the highest AAMR in 1999 (AAMR: 7.38; 95% CI: 5.86 to 9.18), achieved the most notable reduction, reaching an AAMR of 2.09 (95% CI: 1.41 to 2.98) by 2023. This corresponds to an AAPC of − 5.04* (95% CI: − 6.81 to − 3.25). Conversely, Oklahoma experienced a significant post-2010 rise in AAMR, with the highest AAMR of all states in 2023 (AAMR: 2.76; 95% CI: 2.15 to 3.48) (Supplementary Tables S1 and S2). States such as West Virginia, California, Connecticut, and Georgia exhibited pronounced declines of AAMRs in the lung cancer cohort, with AAPCs ≤–4.01. However, several states, including Minnesota (AAPC: − 0.05, *p* = 0.96), Oklahoma (AAPC: − 0.92, *p* = 0.53), and Washington (AAPC: − 1.26, *p* = 0.27), did not demonstrate statistically significant declines in CVD mortality among lung cancer patients, despite significant reductions in overall CVD mortality rates in these states. The decline in CVD mortality was significantly more pronounced in adults with lung cancer than in those with CVD alone in states such as Alabama, California, and Connecticut (Supplementary Table S3).

## Discussion

**Fig. 6:**
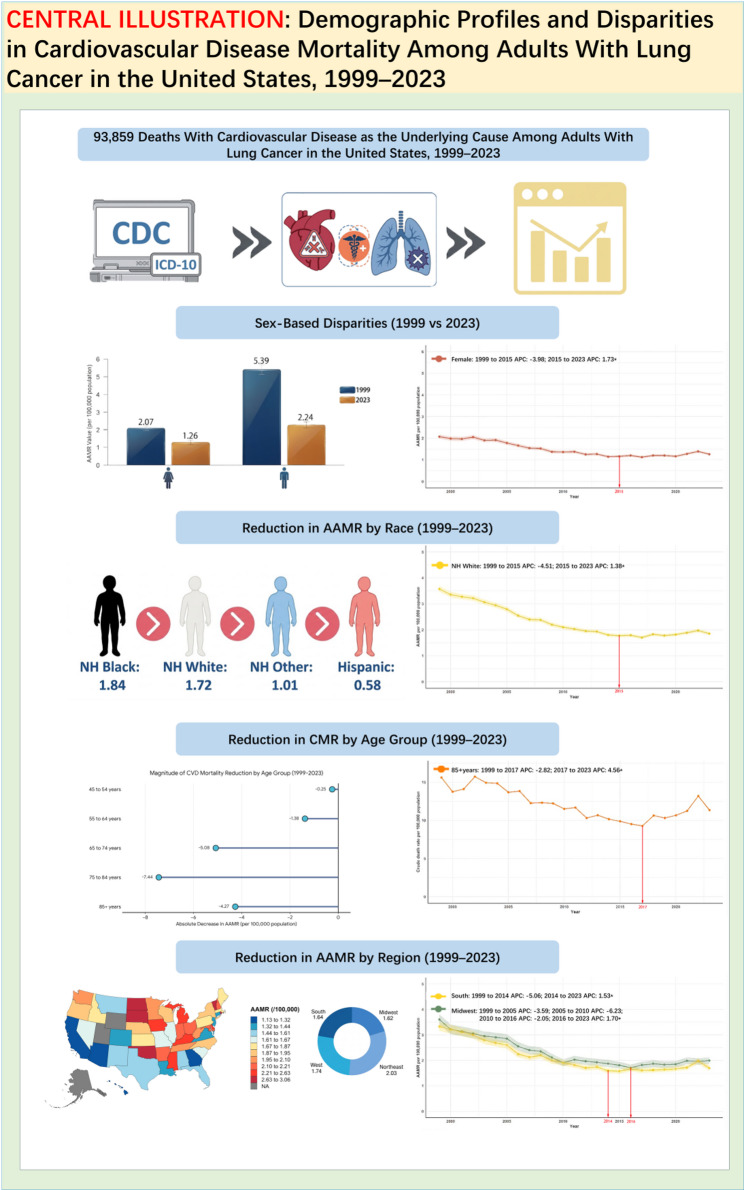
Central figure. Abbreviations: AAMR, age-adjusted mortality rate; APC, annual percent change; CMR, crude mortality rate; NH, non-Hispanic

This nationwide retrospective study utilized the CDC WONDER Multiple Cause of Death database to systematically examine temporal trends in CVD mortality among adults with lung cancer in the US from 1999 to 2023. Notably, in certain subgroups, the long-term decline has either reversed or plateaued in recent years, underscoring important shifts in the CVD mortality burden among lung cancer patients (Fig. 6). A critical consideration in interpreting these findings is the dramatic transformation of the lung cancer therapeutic landscape that began around 2015. The Food and Drug Administration (FDA) approval of nivolumab for advanced NSCLC in 2015 inaugurated the era of ICIs for lung cancer, and ICIs are now a cornerstone of treatment for both advanced and, increasingly, early-stage disease. However, ICI therapy is associated with a distinct spectrum of cardiovascular immune-related adverse events, including fulminant myocarditis, conduction abnormalities, arrhythmias, non-inflammatory heart failure, and accelerated atherosclerosis. The temporal concordance between ICI introduction and the observed inflection in CVD mortality—particularly among females and non-Hispanic Whites—raises the hypothesis that treatment-related cardiotoxicity may contribute to these epidemiological trends. Furthermore, targeted therapies such as osimertinib and ALK inhibitors, also associated with cardiovascular toxicities, have seen expanded use over this period. While this ecological study cannot establish causality, the biological plausibility and temporal alignment warrant urgent investigation in studies with individual-level treatment data.

Between 1999 and 2023, a total of 93,859 deaths were recorded in which CVD was the underlying cause among adults with lung cancer. The AAMR declined from 3.42 to 1.68 per 100,000—a 50.88% reduction. Males consistently had higher absolute CVD mortality rates than females, yet the long-term decline was more pronounced in males. In contrast, among females, this trend reversed significantly after 2015. This sex-specific reversal coincides with the widespread adoption of ICIs, which have brought sex-specific cardiovascular vulnerabilities into sharper focus. Mechanistically, estradiol confers cardioprotection through estrogen receptor α-mediated antioxidant defense [24]. Zhang et al. demonstrated that ICI treatment reduced serum estradiol concentrations to a greater extent in female mice than in male mice and markedly downregulated the cardiac expression of the cardioprotective factors MANF and HSPA5, thereby attenuating the myocardial cell response to immune injury [25]. These preclinical findings align with emerging clinical observations: female patients with advanced NSCLC receiving ICIs face a substantially greater risk of severe symptomatic immune-related adverse events and treatment discontinuation [26]. In keeping with this evidence, female cancer patients receiving immunotherapy face a 49% higher risk of serious adverse events compared with male counterparts [27]. Furthermore, women remain impressively under-represented in ICI randomized controlled trials over the past decade, potentially affecting treatment outcomes for female patients [28].

Racial stratification revealed that CVD mortality declined across all groups from 1999 to 2023, yet the post-2015 reversal was significant among NH White individuals. One potential explanation is the differential uptake of ICIs across racial groups: Surveillance, Epidemiology, and End Results (SEER)-Medicare data demonstrate higher ICI utilization rates among NH-White patients with advanced NSCLC compared with other racial/ethnic groups [29]. Thus, greater exposure to potentially cardiotoxic therapies may partly underlie the observed inflection in this subgroup, though causality cannot be inferred from ecological data.

The NH Black population exhibited the largest absolute decline in AAMR (49% reduction, from 1999 to 2023), consistent with national trends showing the greatest relative decline in cancer mortality among Black males due to decreased smoking initiation among Black teenagers, treatment advances, and earlier detection [30]. Nevertheless, the decline in CVD mortality among NH Black individuals decelerated after 2015, and this group consistently maintained the highest AAMRs throughout most of the study period, highlighting a persistently high disease burden. Prior SEER-based analyses similarly demonstrate higher all-cause and CVD mortality among NH Black cancer patients [31]. Contributing factors include adverse social determinants of health, inadequate insurance coverage, lower income levels, reduced educational attainment, and higher prevalence of traditional cardiovascular risk factors with suboptimal disease management [32–34].

Analysis by age group revealed a concerning upward trend in AAMR for CVD among lung cancer patients aged ≥ 85 years since 2017, contrasting with continued declines in younger cohorts. Potential contributors include later-stage cancer diagnosis, greater comorbidity burden, functional decline, and under-treatment in this vulnerable population [35], compounded by the notable underrepresentation of older adults in clinical trials that define cancer care standards [36].

Regionally, AAMRs declined across all four census regions, yet recent trends diverged. The Northeast experienced sustained decline until 2017 followed by stabilization, whereas the Midwest and South exhibited significant increases after 2016 and 2014, respectively. These patterns may reflect that the Midwest and South regions are comparatively deficient in healthcare resources [37] and that there is a higher CVD burden in rural Southern areas [38]. Factors such as higher exposure to particulate matter pollution, lower levels of education and income, increased rates of obesity and physical inactivity, limited access to healthcare, and higher unemployment rates may disproportionately affect individuals in the South [39]. Inadequate management of cardiovascular risk factors, such as low statin utilization among eligible lung cancer survivors, may further contribute [40].

State-level analyses revealed marked geographic heterogeneity. For example, West Virginia, California, Connecticut, and Georgia experienced pronounced declines in AAMR, whereas Minnesota saw a 64% increase in the number of deaths and Oklahoma experienced a significant post-2010 rise in AAMR.

Overall, the AAMR for CVD among adults with lung cancer decreased by 50.88% from 1999 to 2023, exceeding the 38.43% decline in the general US population (Supplementary Table S5). Despite this progress, recent stagnation or reversal in key subgroups—particularly females, NH Whites, and the oldest old—underscores the need for integrated cardio-oncology care models. Such models should incorporate baseline cardiovascular risk stratification, proactive monitoring for cardiotoxicity, and aggressive management of traditional risk factors within routine oncology practice [41]. Telemedicine may further enhance access to cardiovascular surveillance and coordinated care, particularly in underserved regions [42].

### Limitations

First, lung cancer and CVD diagnoses depend on ICD-10 codes from death certificates, which may be underreported or misreported. This is particularly true for patients with advanced lung cancer, in whom cardiopulmonary failure may be difficult to attribute to a single primary cause. Although the National Center for Health Statistics applied standardized World Health Organization-based coding rules throughout the study period, secular changes in death certification practices, physician awareness of cancer therapy-related cardiotoxicity, and coding behavior may have influenced the observed temporal trends. Misclassification may lead to either underestimation or overestimation of CVD mortality, and this possibility should be considered when interpreting the magnitude of the observed trends. Second, the database does not contain information on cancer stage at diagnosis. As a result, we could not assess whether earlier detection following the 2013 U.S. Preventive Services Task Force low-dose CT screening recommendations [43], together with improving survival, increased the number of patients living long enough to die from competing cardiovascular causes. This survivorship effect may partly contribute to the observed recent plateau or increase in CVD mortality. Third, the racial classifications included in NH Other were adjusted after 2021, which may affect the consistency of long-term comparisons. Finally, this analysis did not calculate the overall AAMR for the entire survey period, but annual AAMR trends from 1999 to 2023 (stratified by variable) are shown in Figs. 1, 2, 3 and 4. Additionally, Table 2 provides the specific AAMRs and 95% CIs for the first and final years of the survey period.

## Conclusion

This study showed concerning shifts in CVD mortality trends among US adults with lung cancer from 1999 to 2023, with recent years characterized by a deceleration or reversal of the previously long-term decline in several subgroups. Pronounced disparities in mortality patterns were observed across categories of sex, race/ethnicity, age group, census region, and state. The factors underlying these patterns are likely multifactorial—including the introduction of immune checkpoint inhibitors and targeted therapies since 2015, which temporally coincides with mortality inflection points in females and Non-Hispanic White individuals. While these ecological findings do not allow for causal inferences, they suggest opportunities for integrated cardio-oncology care and targeted public health strategies. Future research using individual-level treatment data is crucial to establish causality and develop evidence-based preventive measures.

## Supplementary Information

Supplementary Material 1.

Supplementary Material 2.

## Data Availability

All data generated or analyzed during this study are included in this published article and its supplementary information files, which are freely available on the CDC WONDER database. (CDC WONDER --- CDC WONDER, Accessed 1 Oct 2025)

## Abbreviations

CVD: Cardiovascular disease
US: United States
CDC WONDER: Centers for Disease Control and Prevention’s Wide-Ranging Online Data for Epidemiologic Research
AAMR: Age-adjusted mortality rate
CMR: Crude mortality rate
APC: Annual percent change
AAPC: Average annual percent change
NH Black: Non-Hispanic African American or Black
NSCLC: Non-small cell lung cancer
ICIs: Immune checkpoint inhibitors
PD-1: Programmed cell death protein 1
STROBE: Strengthening the Reporting of Observational Studies in Epidemiology
ICD: International Classification of Diseases
CI: Confidence interval
FDA: Food and Drug Administration
SEER: Surveillance, Epidemiology, and End Results
