# Supplementary material for: Trends in cardiovascular mortality among lung cancer patients in the United States: a retrospective study from 1999 to 2023
Source: Cardiooncology. 2026 May 22;12:97. doi: 10.1186/s40959-026-00512-z (PMC13410872; doi:10.1186/s40959-026-00512-z)
Supplement: Supplementary file 1 — Supplementary Material 1. [file 40959_2026_512_MOESM1_ESM.docx]

**Trends in Cardiovascular Mortality Among Lung Cancer Patients in the United States: a retrospective study from 1999 to 2023**

**Yong‐li Chen** **ᵃ,** **Jian-quan Chen ^a^, Lin-wen Zeng ^a^, Jin-yan Wu ^a^,** **Xiao‐xia Qiu ᵇ, Zhen-nan Lin ^ac^, Jie Xiao ^d^, Xing Miao ^a^, Re‐hua Wang ᵃ^,^*, Jian‐cheng Zhang ^a,^***

ᵃ Yong‐li Chen, M.D. Department of Cardiology, Fuzhou University Affiliated Provincial Hospital; Shengli Clinical Medical College of Fujian Medical University; Fujian Provincial Hospital, Fuzhou, Fujian, PR China. [yonglichen2022@163.com](mailto:yonglichen2022@163.com); https://orcid.org/0009-0006-4690-7531.

ᵃ Jian-quan Chen, Ph.D. Department of Cardiology, Fuzhou University Affiliated Provincial Hospital; Shengli Clinical Medical College of Fujian Medical University; Fujian Provincial Hospital, Fuzhou, Fujian, PR China. ccjqemie@163.com.

ᵃ Lin-wen Zeng, Ph.D. Department of Cardiology, Fuzhou University Affiliated Provincial Hospital; Shengli Clinical Medical College of Fujian Medical University; Fujian Provincial Hospital, Fuzhou, Fujian, PR China. zenglinwen18@163.com.

ᵃ Jin-yan Wu, B.S. Department of Cardiology, Fuzhou University Affiliated Provincial Hospital; Shengli Clinical Medical College of Fujian Medical University; Fujian Provincial Hospital, Fuzhou, Fujian, PR China. [410093828@qq.com](mailto:410093828@qq.com).

ᵇ Xiao‐xia Qiu, M.D. Department of Cardiology, Fujian Medical University Union Hospital, Fuzhou, Fujian, PR China. yishu1997@qq.com; https://orcid.org/0000-0003-3613-4476.

ᶜ Zhen-nan Lin, Ph.D. Fujian Provincial Key Laboratory of Cardiovascular Disease, Fujian Cardiovascular Institute, Fujian Provincial Center for Geriatrics, Fujian Clinical Medical Research Center for Cardiovascular Diseases, Fuzhou, Fujian, PR China. linzhennan2019@163.com.

^d^ Jie Xiao, M.D. Department of Ultrasound, the First Affiliated Hospital, Fujian Medical University, Fuzhou, Fujian, PR China; Department of Ultrasound, National Regional Medical Center, Binhai Campus of the First Affiliated Hospital, Fujian Medical University, Fuzhou, Fujian, PR China. [xiaojie1182023@163.com](mailto:xiaojie1182023@163.com).

ᵃ Xing Miao, B.S. Department of Cardiology, Fuzhou University Affiliated Provincial Hospital; Shengli Clinical Medical College of Fujian Medical University; Fujian Provincial Hospital, Fuzhou, Fujian, PR China. [miaoxing126@126.com](mailto:miaoxing126@126.com).

***Corresponding author**: ᵃ Re‐hua Wang, Ph.D. Department of Cardiology, Fuzhou University Affiliated Provincial Hospital; Shengli Clinical Medical College of Fujian Medical University; Fujian Provincial Hospital, Fuzhou, Fujian, PR China. [rehuawang03@163.com](mailto:rehuawang03@163.com), https://orcid.org/0000-0002-2988-1433. ᵃ Jian‐cheng Zhang, Ph.D. Department of Cardiology, Fuzhou University Affiliated Provincial Hospital; Shengli Clinical Medical College of Fujian Medical University; Fujian Provincial Hospital, Fuzhou 350001, Fujian, PR China. E-mail addresses: [fjzhangjiancheng@126.com](mailto:fjzhangjiancheng@126.com); Phone number: +8613696860345; Fax:0591-87532356; https://orcid.org/0000-0002-8848-5748.

**Short title**

Rebound of Cardiovascular Mortality in Lung Cancer

**Disclosures**

The authors declare no competing interests.

**Statement of authorship**

All authors take responsibility for all aspects of the reliability and freedom from bias of the data presented and their discussed interpretation.
