# Supplementary material for: Trends in cardiovascular mortality among lung cancer patients in the United States: a retrospective study from 1999 to 2023
Source: Cardiooncology. 2026 May 22;12:97. doi: 10.1186/s40959-026-00512-z (PMC13410872; doi:10.1186/s40959-026-00512-z)
Supplement: Supplementary file 2 — Supplementary Material 2. [file 40959_2026_512_MOESM2_ESM.docx]

**Supplementary Material**

**Trends in Cardiovascular Mortality Among Lung Cancer Patients in the United States: a retrospective study from 1999 to 2023**

This supplementary material is offered by the authors to enhance readers' understanding of their research outcomes.

| **Supplementary Table S1**  The number of deaths and age-adjusted mortality rates (AAMRs) for cardiovascular disease among adults with lung cancer in the US in 1999 and 2023–Stratified by state. | | | | | | | | | | | | | |  |
| --- | --- | --- | --- | --- | --- | --- | --- | --- | --- | --- | --- | --- | --- | --- |
| State | Deaths | | | | | | | AAMR | | | | | |  |
|  | 1999 | | 2023 | | Percent  Change (%) | | | 1999(95% CI） | | 2023(95% CI） | | Decreased values | |  |
| Alabama | 73 | | 50 | | -31.51 | | | 3.16 (2.48 to 3.98) | | 1.44 (1.06 to 1.91) | | -1.72 | |  |
| Arizona | 51 | | 62 | | 21.57 | | | 1.97 (1.47 to 2.59) | | 1.13 (0.87 to 1.45) | | -0.84 | |  |
| Arkansas | 42 | | 44 | | 4.76 | | | 2.85 (2.06 to 3.85) | | 2.10 (1.52 to 2.81) | | -0.75 | |  |
| California | 531 | | 296 | | -44.26 | | | 3.67 (3.36 to 3.99) | | 1.22 (1.08 to 1.35) | | -2.45 | |  |
| Colorado | 38 | | 46 | | 21.05 | | | 2.24 (1.59 to 3.08) | | 1.33 (0.97 to 1.78) | | -0.91 | |  |
| Connecticut | 92 | | 37 | | -59.78 | | | 4.81 (3.88 to 5.90) | | 1.37 (0.97 to 1.89) | | -3.44 | |  |
| Florida | 292 | | 282 | | -3.42 | | | 2.67 (2.36 to 2.98) | | 1.48 (1.30 to 1.66) | | -1.19 | |  |
| Georgia | 101 | | 79 | | -21.78 | | | 3.14 (2.52 to 3.76) | | 1.21 (0.95 to 1.52) | | -1.93 | |  |
| Illinois | 215 | | 140 | | -34.88 | | | 3.53 (3.06 to 4.00) | | 1.65 (1.37 to 1.92) | | -1.88 | |  |
| Indiana | 112 | | 101 | | -9.82 | | | 3.69 (3.01 to 4.37) | | 2.19 (1.76 to 2.63) | | -1.50 | |  |
| Iowa | 56 | | 54 | | -3.57 | | | 3.23 (2.44 to 4.20) | | 2.42 (1.81 to 3.17) | | -0.81 | |  |
| Kentucky | 62 | | 79 | | 27.42 | | | 3.06 (2.35 to 3.93) | | 2.58 (2.04 to 3.23) | | -0.48 | |  |
| Louisiana | 39 | | 43 | | 10.26 | | | 1.88 (1.34 to 2.57) | | 1.36 (0.98 to 1.84) | | -0.52 | |  |
| Maryland | 107 | | 89 | | -16.82 | | | 4.35 (3.53 to 5.18) | | 2.14 (1.72 to 2.64) | | -2.21 | |  |
| Massachusetts | 106 | | 91 | | -14.15 | | | 3.09 (2.50 to 3.68) | | 1.82 (1.46 to 2.23) | | -1.27 | |  |
| Michigan | 190 | | 159 | | -16.32 | | | 3.89 (3.34 to 4.45) | | 2.21 (1.86 to 2.56) | | -1.68 | |  |
| Minnesota | 50 | | 82 | | 64.00 | | | 2.07 (1.54 to 2.73) | | 2.07 (1.65 to 2.58) | | 0.00 | |  |
| Mississippi | 63 | | 47 | | -25.40 | | | 4.58 (3.52 to 5.86) | | 2.42 (1.77 to 3.23) | | -2.16 | |  |
| Missouri | 107 | | 70 | | -34.58 | | | 3.54 (2.87 to 4.21) | | 1.61 (1.25 to 2.04) | | -1.93 | |  |
| New Jersey | 127 | | 85 | | -33.07 | | | 2.86 (2.36 to 3.35) | | 1.32 (1.05 to 1.63) | | -1.54 | |  |
| New York | 431 | | 264 | | -38.75 | | | 4.37 (3.96 to 4.78) | | 1.87 (1.64 to 2.09) | | -2.50 | |  |
| North Carolina | 156 | | 139 | | -10.90 | | | 4.00 (3.37 to 4.63) | | 1.92 (1.60 to 2.25) | | -2.08 | |  |
| Ohio | 265 | | 179 | | -32.45 | | | 4.40 (3.87 to 4.93) | | 2.17 (1.85 to 2.50) | | -2.23 | |  |
| Oklahoma | 62 | | 71 | | 14.52 | | | 3.41 (2.62 to 4.38) | | 2.76 (2.15 to 3.48) | | -0.65 | |  |
| Oregon | 67 | | 60 | | -10.45 | | | 3.77 (2.92 to 4.78) | | 1.95 (1.49 to 2.52) | | -1.82 | |  |
| Pennsylvania | 253 | | 165 | | -34.78 | | | 3.37 (2.95 to 3.79) | | 1.67 (1.42 to 1.93) | | -1.70 | |  |
| South Carolina | 65 | | 65 | | 0.00 | | | 3.41 (2.62 to 4.35) | | 1.63 (1.26 to 2.09) | | -1.78 | |  |
| Tennessee | 93 | | 100 | | 7.53 | | | 3.27 (2.64 to 4.01) | | 2.08 (1.67 to 2.49) | | -1.19 | |  |
| Texas | 284 | | 236 | | -16.90 | | | 3.42 (3.02 to 3.81) | | 1.49 (1.30 to 1.68) | | -1.93 | |  |
| Virginia | 115 | | 83 | | -27.83 | | | 3.61 (2.95 to 4.27) | | 1.43 (1.14 to 1.78) | | -2.18 | |  |
| Washington | 83 | | 98 | | 18.07 | | | 3.07 (2.45 to 3.81) | | 1.95 (1.58 to 2.39) | | -1.12 | |  |
| West Virginia | 81 | | 31 | | -61.73 | | | 7.38 (5.86 to 9.18) | | 2.09 (1.41 to 2.98) | | -5.29# | |  |
| Wisconsin | 90 | | 79 | | -12.22 | | | 3.19 (2.56 to 3.92) | | 1.90 (1.50 to 2.37) | | -1.29 | |  |
| Abbreviations: AAMR, age-adjusted mortality rate; CI, confidence interval.  # The state with the largest drop in AAMR for cardiovascular disease in lung cancer patients. | | | | | | | | | | | | | |  |
| **Supplementary Table S2** | | | | | | | | | | | | | | |
| Annual percentage changes (APCs) and average annual percentage changes (AAPCs) in cardiovascular disease mortality among adults with lung cancer in the US from 1999 to 2023–Stratified by state. | | | | | | | | | | | | | | |
| State | | Trend Segment | | Start | | End | APC (95%CI) | | P-value | | AAPC (95% CI） | | P-value | |
| Alabama | | 1 | | 1999 | | 2016 | -5.88*(-6.98to-4.76) | | ＜0.000001 | | -3.51*(-5.02to-1.98) | | 0.000009 | |
|  |  | 2 | | 2016 | | 2023 | 2.48(-2.49to7.71) | | 0.316085 | |  |  |  |  |
| Arizona | | 1 | | 1999 | | 2023 | -2.08*(-2.78to-1.38) | | 0.000003 | | -2.08*(-2.78to-1.38) | | 0.000003 | |
| Arkansas | | 1 | | 1999 | | 2014 | -3.68*(-5.27to-2.07) | | 0.000136 | | -1.50(-3.04to0.07) | | 0.061803 | |
|  |  | 2 | | 2014 | | 2023 | 2.26(-1.29to5.93) | | 0.201910 | |  |  |  |  |
| California | | 1 | | 1999 | | 2002 | -1.35(-7.28to4.97) | | 0.650241 | | -4.36*(-5.33to-3.38) | | <0.000001 | |
|  |  | 2 | | 2002 | | 2015 | -6.92*(-7.73to-6.11) | | ＜0.000001 | |  |  |  |  |
|  |  | 3 | | 2015 | | 2023 | -1.20(-2.99to0.63) | | 0.182948 | |  |  |  |  |
| Colorado | | 1 | | 1999 | | 2023 | -2.36*(-3.72to-0.99) | | 0.001773 | | -2.36*(-3.72to-0.99) | | 0.001773 | |
| Connecticut | | 1 | | 1999 | | 2023 | -4.37*(-5.17to-3.56) | | ＜0.000001 | | -4.37*(-5.17to-3.56) | | <0.000001 | |
| Florida | | 1 | | 1999 | | 2014 | -5.00*(-6.17to-3.81) | | ＜0.000001 | | -1.99*(-3.13to-0.84) | | 0.000739 | |
|  |  | 2 | | 2014 | | 2023 | 3.24*(0.60to5.95) | | 0.018339 | |  |  |  |  |
| Georgia | | 1 | | 1999 | | 2016 | -6.26*(-7.23to-5.29) | | ＜0.000001 | | -4.01*(-5.27to-2.73) | | <0.000001 | |
|  |  | 2 | | 2016 | | 2023 | 1.71(-2.40to5.99) | | 0.402319 | |  |  |  |  |
| Illinois | | 1 | | 1999 | | 2019 | -4.21*(-4.76to-3.67) | | ＜0.000001 | | -2.87*(-4.02to-1.72) | | 0.000001 | |
|  |  | 2 | | 2019 | | 2023 | 4.11(-2.93to11.66) | | 0.244168 | |  |  |  |  |
| Indiana | | 1 | | 1999 | | 2009 | -5.63*(-8.73to-2.42) | | 0.001720 | | -2.11*(-3.79to-0.39) | | 0.016370 | |
|  |  | 2 | | 2009 | | 2023 | 0.49(-1.58to2.61) | | 0.626621 | |  |  |  |  |
| Iowa | | 1 | | 1999 | | 2007 | -7.18*(-12.29to-1.77) | | 0.012573 | | -2.13 (-4.23to0.01) | | 0.051411 | |
|  |  | 2 | | 2007 | | 2023 | 0.49(-1.49to2.51) | | 0.614923 | |  |  |  |  |
| Kentucky | | 1 | | 1999 | | 2014 | -4.22*(-5.80to-2.61) | | 0.000028 | | -1.16 (-2.69to0.39) | | 0.142005 | |
|  |  | 2 | | 2014 | | 2023 | 4.15*(0.62to7.80) | | 0.023197 | |  |  |  |  |
| Louisiana | | 1 | | 1999 | | 2023 | -1.23*(-2.37to-0.07) | | 0.038340 | | -1.23*(-2.37to-0.07) | | 0.038340 | |
| Maryland | | 1 | | 1999 | | 2008 | -3.78*(-6.19to-1.31) | | 0.005234 | | -2.64*(-4.97to-0.26) | | 0.029669 | |
|  |  | 2 | | 2008 | | 2013 | -11.40*(-20.17to-1.66) | | 0.025487 | |  |  |  |  |
|  |  | 3 | | 2013 | | 2023 | 3.14*(0.53to5.81) | | 0.020770 | |  |  |  |  |
| Massachusetts | | 1 | | 1999 | | 2023 | -2.81*(-3.46to-2.16) | | ＜0.000001 | | -2.81*(-3.46to-2.16) | | <0.000001 | |
| Michigan | | 1 | | 1999 | | 2015 | -5.13*(-5.97to-4.28) | | ＜0.000001 | | -2.83*(-3.81to-1.85) | | <0.000001 | |
|  |  | 2 | | 2015 | | 2023 | 1.92(-0.77to4.68) | | 0.154029 | |  |  |  |  |
| Minnesota | | 1 | | 1999 | | 2013 | -3.03*(-5.41to-0.59) | | 0.017689 | | -0.05 (-1.96to1.89) | | 0.959062 | |
|  |  | 2 | | 2013 | | 2023 | 4.28*(0.71to7.97) | | 0.020839 | |  |  |  |  |
| Mississippi | | 1 | | 1999 | | 2016 | -5.38*(-7.04to-3.69) | | 0.000002 | | -1.92 (-4.17to0.38) | | 0.100497 | |
|  |  | 2 | | 2016 | | 2023 | 7.01(-0.52to15.10) | | 0.066879 | |  |  |  |  |
| Missouri | | 1 | | 1999 | | 2019 | -5.08*(-6.00to-4.14) | | ＜0.000001 | | -2.88*(-4.90to-0.82) | | 0.006320 | |
|  |  | 2 | | 2019 | | 2023 | 8.89(-3.87to23.34) | | 0.169491 | |  |  |  |  |
| New Jersey | | 1 | | 1999 | | 2023 | -2.76*(-3.28to-2.24) | | ＜0.000001 | | -2.76*(-3.28to-2.24) | | <0.000001 | |
| New York | | 1 | | 1999 | | 2002 | 2.81(-4.48to10.64) | | 0.437566 | | -3.16*(-4.34to-1.96) | | <0.000001 | |
|  |  | 2 | | 2002 | | 2017 | -5.22*(-5.96to-4.47) | | ＜0.000001 | |  |  |  |  |
|  |  | 3 | | 2017 | | 2023 | -0.82(-4.00to2.46) | | 0.600855 | |  |  |  |  |
| North Carolina | | 1 | | 1999 | | 2017 | -4.91*(-5.79to-4.03) | | ＜0.000001 | | -2.52*(-3.76to-1.26) | | 0.000094 | |
|  |  | 2 | | 2017 | | 2023 | 5.03*(0.22to10.07) | | 0.041174 | |  |  |  |  |
| Ohio | | 1 | | 1999 | | 2016 | -4.18*(-4.96to-3.39) | | ＜0.000001 | | -2.57*(-3.62to-1.51) | | 0.000002 | |
|  |  | 2 | | 2016 | | 2023 | 1.44(-1.95to4.95) | | 0.390306 | |  |  |  |  |
| Oklahoma | | 1 | | 1999 | | 2004 | 2.62(-5.64to11.59) | | 0.524566 | | -0.92 (-3.76to2.01) | | 0.534401 | |
|  |  | 2 | | 2004 | | 2010 | -10.53*(-18.45to-1.85) | | 0.021336 | |  |  |  |  |
|  |  | 3 | | 2010 | | 2023 | 2.47*(0.26to4.73) | | 0.030514 | |  |  |  |  |
| Oregon | | 1 | | 1999 | | 2016 | -3.82*(-5.01to-2.61) | | 0.000002 | | -1.42 (-2.91to0.09) | | 0.065443 | |
|  | | 2 | | 2016 | | 2023 | 4.65(-0.10to9.64) | | 0.054684 | |  | |  | |
| Pennsylvania | | 1 | | 1999 | | 2017 | -4.32*(-4.98to-3.66) | | ＜0.000001 | | -2.38*(-3.39to-1.36) | | 0.000006 | |
|  |  | 2 | | 2017 | | 2023 | 3.69(-0.29to7.83) | | 0.067455 | |  |  |  |  |
| South Carolina | | 1 | | 1999 | | 2023 | -2.64*(-3.58to-1.69) | | 0.000008 | | -2.64*(-3.58to-1.69) | | 0.000008 | |
| Tennessee | | 1 | | 1999 | | 2018 | -3.26*(-4.20to-2.31) | | 0.000001 | | -1.60*(-3.15to-0.02) | | 0.047052 | |
|  |  | 2 | | 2018 | | 2023 | 4.99(-2.30to12.83) | | 0.173440 | |  |  |  |  |
| Texas | | 1 | | 1999 | | 2012 | -4.87*(-5.99to-3.75) | | ＜0.000001 | | -3.34*(-4.21to-2.46) | | <0.000001 | |
|  |  | 2 | | 2012 | | 2023 | -1.50(-3.04to0.07) | | 0.060745 | |  |  |  |  |
| Virginia | | 1 | | 1999 | | 2014 | -6.27*(-7.35to-5.17) | | ＜0.000001 | | -3.60*(-4.73to-2.46) | | <0.000001 | |
|  |  | 2 | | 2014 | | 2023 | 1.02(-1.69to3.80) | | 0.444234 | |  |  |  |  |
| Washington | | 1 | | 1999 | | 2010 | -0.99(-3.07to1.13) | | 0.333916 | | -1.26 (-3.46to0.98) | | 0.268751 | |
|  |  | 2 | | 2010 | | 2016 | -8.46*(-14.97to-1.46) | | 0.021624 | |  |  |  |  |
|  |  | 3 | | 2016 | | 2023 | 4.92*(0.59to9.44) | | 0.028005 | |  |  |  |  |
| West Virginia | | 1 | | 1999 | | 2005 | -11.19*(-16.65to-5.37) | | 0.000884 | | -5.04*(-6.81to-3.25) | | <0.000001 | |
|  |  | 2 | | 2005 | | 2023 | -2.90*(-4.45to-1.33) | | 0.001085 | |  |  |  |  |
| Wisconsin | | 1 | | 1999 | | 2013 | -4.85*(-6.72to-2.94) | | 0.000042 | | -1.19 (-2.79to0.43) | | 0.148706 | |
|  |  | 2 | | 2013 | | 2023 | 4.16*(1.00to7.43) | | 0.012123 | |  |  |  |  |
| Abbreviations: APC, annual percent change; CI, confidence interval; AAPC, average annual percent change. | | | | | | | | | | | | | | |
| * Shows that the APC or AAPC significantly deviates from zero at the alpha = 0.05 level. | | | | | | | | | | | | | | |

| **Supplementary Table S3** | | | | | | |
| --- | --- | --- | --- | --- | --- | --- |
| Evaluating the differences in cardiovascular disease mortality between adults with lung cancer and the general US population from 1999 to 2023–Stratified by state. | | | | | | |
| State | Cardiovascular Disease in Patients with Lung cancer | |  | Overall Cardiovascular Disease-Related Mortality Rates | | P-value for AAPC Comparison |
|  | AAPC (95%CI) | P-Value |  | AAPC (95%CI) | P-value |  |
| Alabama# | -3.51*(-5.02to-1.98) | 0.000009 |  | -1.26*(-1.88to-0.64) | 0.000068 | 0.007222 |
| Arizona | -2.08*(-2.78to-1.38) | 0.000003 |  | -1.70*(-2.09to-1.30) | <0.000001 | 0.354109 |
| Arkansas | -1.50(-3.04to0.07) | 0.061803 |  | -1.36*(-1.59to-1.14) | <0.000001 | 0.861359 |
| California# | -4.36*(-5.33to-3.38) | <0.000001 |  | -2.26*(-2.52to-2.01) | <0.000001 | 0.000044 |
| Colorado | -2.36*(-3.72to-0.99) | 0.001773 |  | -1.96*(-2.41to-1.51) | <0.000001 | 0.585423 |
| Connecticut# | -4.37*(-5.17to-3.56) | <0.000001 |  | -2.44*(-2.61to-2.26) | <0.000001 | 0.000004 |
| Florida | -1.99*(-3.13to-0.84) | 0.000739 |  | -2.02*(-2.27to-1.77) | <0.000001 | 0.959986 |
| Georgia# | -4.01*(-5.27to-2.73) | <0.000001 |  | -2.17*(-2.92to-1.41) | <0.000001 | 0.014650 |
| Illinois | -2.87*(-4.02to-1.72) | 0.000001 |  | -2.13*(-2.73to-1.51) | <0.000001 | 0.265203 |
| Indiana | -2.11*(-3.79to-0.39) | 0.016370 |  | -1.84*(-2.14to-1.53) | <0.000001 | 0.759299 |
| Iowa | -2.13 (-4.23to0.01) | 0.051411 |  | -1.49*(-1.88to-1.09) | <0.000001 | 0.560777 |
| Kentucky | -1.16 (-2.69to0.39) | 0.142005 |  | -2.04*(-2.74to-1.33) | <0.000001 | 0.308506 |
| Louisiana | -1.23*(-2.37to-0.07) | 0.038340 |  | -1.41*(-1.71to-1.11) | <0.000001 | 0.766582 |
| Maryland | -2.64*(-4.97to-0.26) | 0.029669 |  | -2.27*(-2.92to-1.62) | <0.000001 | 0.766588 |
| Massachusetts | -2.81*(-3.46to-2.16) | <0.000001 |  | -2.48*(-2.68to-2.29) | <0.000001 | 0.340534 |
| Michigan# | -2.83*(-3.81to-1.85) | <0.000001 |  | -1.63*(-2.15to-1.12) | <0.000001 | 0.033628 |
| Minnesota | -0.05 (-1.96to1.89) | 0.959062 |  | -1.88*(-2.21to-1.55) | <0.000001 | 0.066286 |
| Mississippi | -1.92 (-4.17to0.38) | 0.100497 |  | -1.59*(-2.48to-0.69) | 0.000529 | 0.791340 |
| Missouri | -2.88*(-4.90to-0.82) | 0.006320 |  | -1.94*(-2.18to-1.71) | <0.000001 | 0.369610 |
| New Jersey | -2.76*(-3.28to-2.24) | <0.000001 |  | -2.54*(-3.20to-1.87) | <0.000001 | 0.609493 |
| New York | -3.16*(-4.34to-1.96) | <0.000001 |  | -2.57*(-2.83to-2.30) | <0.000001 | 0.342860 |
| North Carolina | -2.52*(-3.76to-1.26) | 0.000094 |  | -2.00*(-2.44to-1.55) | <0.000001 | 0.442406 |
| Ohio | -2.57*(-3.62to-1.51) | 0.000002 |  | -1.73*(-2.36to-1.08) | <0.000001 | 0.182120 |
| Oklahoma | -0.92 (-3.76to2.01) | 0.534401 |  | -1.41*(-1.76to-1.06) | <0.000001 | 0.741044 |
| Oregon | -1.42 (-2.91to0.09) | 0.065443 |  | -1.50*(-2.24to-0.75) | 0.000095 | 0.925409 |
| Pennsylvania | -2.38*(-3.39to-1.36) | 0.000006 |  | -2.07*(-2.27to-1.87) | <0.000001 | 0.556984 |
| South Carolina | -2.64*(-3.58to-1.69) | 0.000008 |  | -2.05*(-2.39to-1.70) | <0.000001 | 0.250352 |
| Tennessee | -1.60*(-3.15to-0.02) | 0.047052 |  | -1.57*(-2.37to-0.77) | 0.000139 | 0.973312 |
| Texas# | -3.34*(-4.21to-2.46) | <0.000001 |  | -2.07*(-2.35to-1.80) | <0.000001 | 0.006649 |
| Virginia# | -3.60*(-4.73to-2.46) | <0.000001 |  | -2.06*(-2.66to-1.47) | <0.000001 | 0.018505 |
| Washington | -1.26 (-3.46to0.98) | 0.268751 |  | -2.06*(-2.34to-1.78) | <0.000001 | 0.483456 |
| West Virginia# | -5.04*(-6.81to-3.25) | <0.000001 |  | -1.76*(-2.08to-1.44) | <0.000001 | 0.000378 |
| Wisconsin | -1.19 (-2.79to0.43) | 0.148706 |  | -1.72*(-1.98to-1.45) | <0.000001 | 0.524352 |
| Abbreviations: AAPC, average annual percent change; CI, confidence interval. | | | | | | |
| a Crude Mortality Rates are reported instead of age-adjusted mortality rates for Age groups. | | | | | | |
| * Shows that the AAPC significantly deviates from zero at the alpha = 0.05 level. | | | | | | |
| # Indicates that the CVD mortality downward trend in adults with lung cancer differed significantly from that of CVD alone at the alpha= 0.05 level. | | | | | | |

**Supplementary Table S4：**

Number of cardiovascular disease deaths among lung cancer patients and general population in the US from 1999 to 2023

| **Number of Cardiovascular Disease Deaths among Lung Cancer Patients** | | |
| --- | --- | --- |
| Variable | Total deaths | Total Population |
| **Sex** | 93,859 | 4,106,502,872 |
| Female | 36,861 | 2,148,922,015 |
| Male | 56,998 | 1,957,580,857 |
| **Age Groups** | 93,607 | 4,106,502,872 |
| 35 to 44 years | * | 1,062,777,200 |
| 45 to 54 years | 2,330 | 1,049,190,082 |
| 55 to 64 years | 11,879 | 893,167,759 |
| 65 to 74 years | 28,306 | 612,598,186 |
| 75 to 84 years | 34,669 | 350,599,150 |
| 85+ years | 16,423 | 138,170,495 |
| **Number of Cardiovascular Disease Deaths among general population** | | |
| Variable | Total deaths | Total Population |
| Both | 21,027,588 | 3,043,725,672 |
| Female | 10,712,363 | 1,616,064,406 |
| Male | 10,315,225 | 1,427,661,266 |
| *Data for the 35 to 44 age group were excluded due to unreliable data (defined as mortality estimates based on less than 20 deaths in a given year). | | |

**Supplementary Table S5:**

Demographic characteristics of deaths and age-adjusted mortality rates (AAMRs) for cardiovascular disease in adults among general US population in 1999 and 2023

| Characteristic | Deaths | | | AAMR | |
| --- | --- | --- | --- | --- | --- |
|  | 1999 | 2023 | Percent Change (%) | 1999(95% CI） | 2023(95% CI） |
| **Sex** | | | | | |
| Both | 930,439 | 894,194 | -3.90 | 982.15 (980.16 to 984.15) | 604.69 (603.42 to 605.95) |
| Female | 502,552 | 425,791 | -15.27 | 838.01 (835.68 to 840.34) | 501.94 (500.42 to 503.46) |
| Male | 427,887 | 468,403 | 9.47 | 1174.65 (1171.05 to 1178.25) | 726.43 (724.30 to 728.55) |
| **Census Region** | | | | | |
| Midwest | 228,676 | 200,154 | -12.47 | 998.32 (994.22 to 1002.41) | 641.69 (638.85 to 644.53) |
| Northeast | 196,376 | 150,568 | -23.33 | 969.47 (965.18 to 973.76) | 542.62 (539.85 to 545.38) |
| South | 336,135 | 361,460 | 7.53 | 1017.17 (1013.72 to 1020.61) | 646.31 (644.18 to 648.44) |
| West | 169,252 | 182,012 | 7.54 | 912.67 (908.32 to 917.02) | 549.14 (546.60 to 551.68) |
| **Race** | | | | | |
| Hispanic | 31,992 | 63,313 | 97.90 | 757.05 (748.44 to 765.66) | 434.42 (430.93 to 437.90) |
| NH Black | 97,646 | 113,344 | 16.08 | 1250.86 (1242.94 to 1258.78) | 783.00 (778.31 to 787.69) |
| NH Other | 15,278 | 35,018 | 129.21 | 658.11 (647.17 to 669.04) | 342.13 (338.52 to 345.74) |
| NH White | 782,451 | 679,375 | -13.17 | 970.20 (968.05 to 972.35) | 622.97 (621.47 to 624.47) |
| **State** | | | | | |
| Alabama | 16,917 | 18,532 | 9.55 | 1095.99 (1079.46 to 1112.52) | 811.68 (799.83 to 823.53) |
| Alaska | 741 | 1,192 | 60.86 | 802.26 (739.75 to 864.78) | 499.19 (469.29 to 529.09) |
| Arizona | 14,075 | 19,334 | 37.36 | 836.83 (822.93 to 850.72) | 543.72 (535.98 to 551.46) |
| Arkansas | 10,958 | 10,779 | -1.63 | 1082.46 (1062.18 to 1102.75) | 788.50 (773.44 to 803.57) |
| California | 94,222 | 89,709 | -4.79 | 972.67 (966.45 to 978.88) | 551.79 (548.16 to 555.43) |
| Colorado | 8,987 | 10,916 | 21.46 | 800.98 (784.36 to 817.60) | 490.07 (480.75 to 499.39) |
| Connecticut | 11,625 | 8,718 | -25.01 | 869.35 (853.52 to 885.18) | 485.76 (475.49 to 496.04) |
| Delaware | 2,435 | 3,262 | 33.96 | 937.68 (900.27 to 975.10) | 651.43 (628.61 to 674.24) |
| District of Columbia | 2,036 | 1,381 | -32.17 | 1049.17 (1003.60 to 1094.75) | 582.67 (551.75 to 613.59) |
| Florida | 64,880 | 70,404 | 8.51 | 888.40 (881.52 to 895.27) | 552.85 (548.72 to 556.98) |
| Georgia | 22,845 | 27,505 | 20.40 | 1069.34 (1055.39 to 1083.28) | 657.70 (649.79 to 665.61) |
| Hawaii | 3,300 | 3,818 | 15.70 | 793.66 (766.52 to 820.81) | 477.88 (462.39 to 493.36) |
| Idaho | 3,449 | 4,357 | 26.33 | 864.46 (835.61 to 893.32) | 552.80 (536.14 to 569.45) |
| Illinois | 42,568 | 34,352 | -19.30 | 1022.06 (1012.35 to 1031.77) | 612.67 (606.13 to 619.20) |
| Indiana | 21,624 | 19,587 | -9.42 | 1053.10 (1039.06 to 1067.14) | 671.05 (661.55 to 680.55) |
| Iowa | 11,720 | 9,775 | -16.60 | 917.20 (900.46 to 933.94) | 637.66 (624.86 to 650.46) |
| Kansas | 9,491 | 8,170 | -13.92 | 919.35 (900.75 to 937.94) | 630.71 (616.90 to 644.51) |
| Kentucky | 15,278 | 13,760 | -9.94 | 1129.54 (1111.61 to 1147.47) | 698.65 (686.79 to 710.50) |
| Louisiana | 15,100 | 14,704 | -2.62 | 1097.44 (1079.91 to 1114.98) | 762.69 (750.16 to 775.21) |
| Maine | 4,515 | 4,445 | -1.55 | 902.56 (876.22 to 928.89) | 582.32 (564.93 to 599.70) |
| Maryland | 15,469 | 15,666 | 1.27 | 962.02 (946.81 to 977.23) | 577.72 (568.60 to 586.85) |
| Massachusetts | 20,413 | 15,334 | -24.88 | 839.69 (828.15 to 851.23) | 468.23 (460.76 to 475.70) |
| Michigan | 35,110 | 33,710 | -3.99 | 1068.17 (1056.99 to 1079.35) | 728.14 (720.27 to 736.02) |
| Minnesota | 13,448 | 12,349 | -8.17 | 776.75 (763.58 to 789.93) | 475.90 (467.44 to 484.37) |
| Mississippi | 11,361 | 10,830 | -4.67 | 1213.80 (1191.47 to 1236.14) | 850.57 (834.31 to 866.82) |
| Missouri | 22,758 | 18,905 | -16.93 | 1087.61 (1073.47 to 1101.76) | 666.23 (656.62 to 675.84) |
| Montana | 2,786 | 3,074 | 10.34 | 835.36 (804.34 to 866.38) | 568.90 (548.44 to 589.35) |
| Nebraska | 6,013 | 5,123 | -14.80 | 894.16 (871.42 to 916.89) | 600.38 (583.79 to 616.97) |
| Nevada | 5,258 | 8,779 | 66.96 | 991.87 (964.15 to 1019.60) | 660.32 (646.23 to 674.41) |
| New Hampshire | 3,606 | 3,827 | 6.13 | 891.72 (862.60 to 920.84) | 547.09 (529.50 to 564.68) |
| New Jersey | 28,723 | 22,452 | -21.83 | 949.13 (938.16 to 960.11) | 525.79 (518.87 to 532.71) |
| New Mexico | 4,482 | 5,920 | 32.08 | 817.04 (793.04 to 841.04) | 594.11 (578.73 to 609.49) |
| New York | 69,465 | 51,330 | -26.11 | 1034.60 (1026.91 to 1042.30) | 540.97 (536.25 to 545.69) |
| North Carolina | 25,827 | 28,924 | 11.99 | 1014.26 (1001.86 to 1026.67) | 626.17 (618.86 to 633.49) |
| North Dakota | 2,482 | 1,942 | -21.76 | 894.72 (859.11 to 930.33) | 555.10 (529.93 to 580.26) |
| Ohio | 42,284 | 37,260 | -11.88 | 1043.38 (1033.43 to 1053.33) | 688.76 (681.68 to 695.83) |
| Oklahoma | 14,417 | 13,994 | -2.93 | 1158.23 (1139.31 to 1177.15) | 837.95 (823.90 to 851.99) |
| Oregon | 10,693 | 11,085 | 3.67 | 866.13 (849.70 to 882.55) | 571.45 (560.67 to 582.22) |
| Pennsylvania | 52,457 | 39,580 | -24.55 | 1009.72 (1001.06 to 1018.38) | 603.51 (597.50 to 609.52) |
| Rhode Island | 3,798 | 3,075 | -19.04 | 878.32 (850.27 to 906.37) | 565.66 (545.49 to 585.84) |
| South Carolina | 13,419 | 15,915 | 18.60 | 1063.30 (1045.20 to 1081.39) | 654.24 (643.86 to 664.62) |
| South Dakota | 2,660 | 2,323 | -12.67 | 853.18 (820.49 to 885.88) | 559.63 (536.46 to 582.79) |
| Tennessee | 21,009 | 22,813 | 8.59 | 1111.71 (1096.66 to 1126.76) | 758.27 (748.27 to 768.27) |
| Texas | 55,624 | 64,348 | 15.68 | 1004.30 (995.93 to 1012.66) | 618.12 (613.27 to 622.96) |
| Utah | 3,839 | 5,562 | 44.88 | 759.43 (735.36 to 783.49) | 574.23 (558.91 to 589.55) |
| Vermont | 1,774 | 1,807 | 1.86 | 825.06 (786.63 to 863.48) | 542.41 (517.02 to 567.80) |
| Virginia | 20,083 | 21,970 | 9.40 | 953.00 (939.77 to 966.23) | 591.03 (583.14 to 598.92) |
| Washington | 16,077 | 16,850 | 4.81 | 867.37 (853.95 to 880.78) | 527.00 (518.95 to 535.04) |
| West Virginia | 8,477 | 6,673 | -21.28 | 1154.61 (1130.01 to 1179.21) | 729.55 (711.77 to 747.34) |
| Wisconsin | 18,518 | 16,658 | -10.04 | 933.41 (919.94 to 946.87) | 609.24 (599.88 to 618.60) |
| Wyoming | 1,343 | 1,416 | 5.44 | 874.62 (827.75 to 921.48) | 541.28 (512.60 to 569.95) |
| **Age Groups^a^** | | | | | |
| 45 to 54 years | 42,890 | 39,433 | -8.06 | 117.26 (116.15 to 118.37) | 97.38 (96.42 to 98.34) |
| 55 to 64 years | 78,278 | 101,777 | 30.02 | 329.20 (326.90 to 331.51) | 243.17 (241.68 to 244.66) |
| 65 to 74 years | 164,120 | 177,409 | 8.10 | 891.04 (886.73 to 895.35) | 511.48 (509.10 to 513.86) |
| 75 to 84 years | 303,669 | 241,568 | -20.45 | 2484.02 (2475.18 to 2492.85) | 1315.15 (1309.91 to 1320.39) |
| 85+ years | 341,482 | 334,007 | -2.19 | 8220.52 (8192.95 to 8248.10) | 5391.58 (5373.29 to 5409.86) |
| Abbreviations: CI, confidence interval; AAMR, Age-adjusted mortality rate; AAPC, average annual percent change. | | | | | |
| ^a^ Crude Mortality Rate is used for analysis instead of age-adjusted mortality rates for Age. | | | | | |
